# Supplementary material for: Participatory Action Research for Tackling Distress and Burnout in Young Medical Researchers: Normative Beliefs before and during the Greek Financial Crisis
Source: Int J Environ Res Public Health. 2022 Aug 23;19(17):10467. doi: 10.3390/ijerph191710467 (PMC9517749; doi:10.3390/ijerph191710467)
Supplement: Supplementary file 1 [file ijerph-19-10467-s001.zip › ijerph-1848212-supplementary.pdf]

Table S1. Theoretical framework: NPT sensitizing questions for participatory interventions for tackling distress/burnout in young medical researchers

NPT constructs	Questions for interventions
**1.Coherence**	How do you conceptualize (make sense of) participatory interventions for tackling distress/burnout?
1.1 Differentiation	Do you feel ready to change your risk behaviors towards tackling distress/burnout syndrome?
1.2 Communal specification	Can you jointly with your collaborators build up a shared understanding of the aims, objectives and expected benefits of a participatory intervention for tackling distress/burnout?
1.3 Individual specification	Which is the work that the implementation of a participatory intervention for tackling distress/burnout would create in your routine work?
1.4 Internalization	Which are the potential benefits, levers and barriers of participating in a participatory intervention for tackling distress/burnout?
**2.Cognitive participation**	
2.1 Initiation	Are you ready and willing to participate to an intervention aiming to tackle distress/burnout syndrome?
2.2 Enrolment	Do you, other young medical researchers and your seniors have the capacity and willingness to organize yourselves to collectively contribute to the work involved in implementing participatory interventions to tackle distress/burnout?
2.3 Legitimation	Do you believe that you and other young medical researchers have the right to be involved in participatory interventions to tackle distress/burnout?
2.4 Activation	Do you feel ready to sustain and continuously behave in line with a behavioral change intervention to tackle distress/burnout?

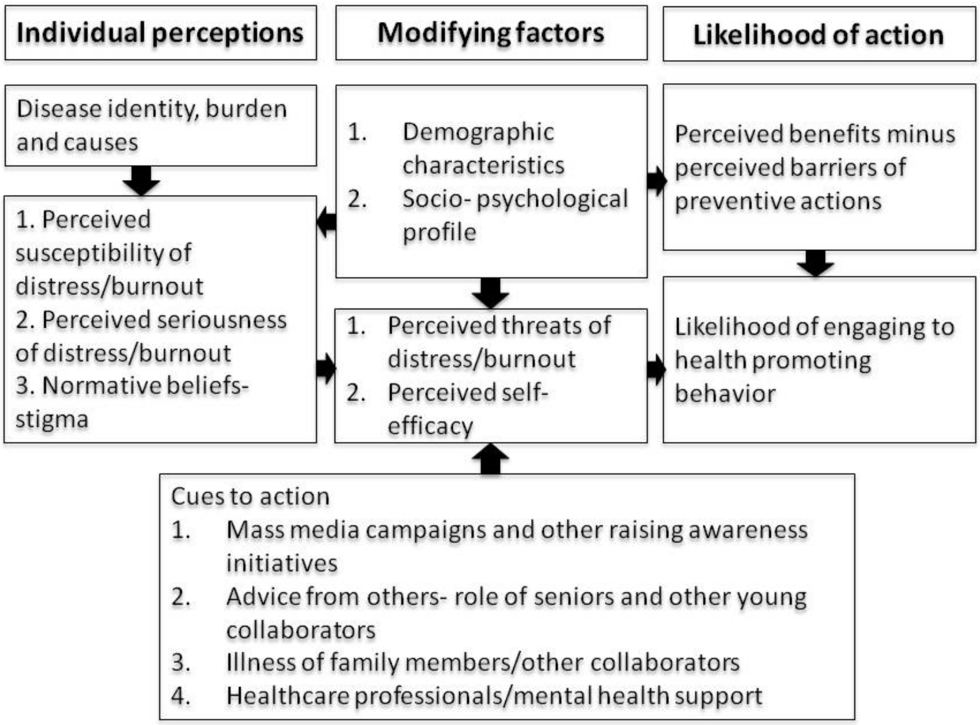

Figure S1. Theoretical framework: HPM questions for assessing individual perceptions, modifying factors and likelihood of action related to tackling distress/burnout
